# Protein Binding Site Prediction by Combining Hidden Markov Support Vector Machine and Profile-Based Propensities

**DOI:** 10.1155/2014/464093

**Published:** 2014-07-14

**Authors:** Bin Liu, Bingquan Liu, Fule Liu, Xiaolong Wang

**Affiliations:** ^1^School of Computer Science and Technology, Harbin Institute of Technology Shenzhen Graduate School, Shenzhen, Guangdong 518055, China; ^2^Key Laboratory of Network Oriented Intelligent Computation, Harbin Institute of Technology Shenzhen Graduate School, Shenzhen, Guangdong 518055, China; ^3^School of Computer Science and Technology, Harbin Institute of Technology, Harbin, Heilongjiang 150001, China

## Abstract

Identification of protein binding sites is critical for studying the function of the proteins. In this paper, we proposed a method for protein binding site prediction, which combined the order profile propensities and hidden Markov support vector machine (HM-SVM). This method employed the sequential labeling technique to the field of protein binding site prediction. The input features of HM-SVM include the profile-based propensities, the Position-Specific Score Matrix (PSSM), and Accessible Surface Area (ASA). When tested on different data sets, the proposed method showed promising results, and outperformed some closely relative methods by more than 10% in terms of AUC.

## 1. Introduction

Prediction of protein binding sites provides valuable information for studying the function of proteins. The most efficient approaches are the computational methods. By using these approaches, the functionally important amino acid residues can be identified [1].

These computational methods used different features extracted from protein sequences, PSSM, or structure information. Hydrophobic and polar residues tend to occur in protein binding regions [2, 3]. The conservation scores of amino acid are often used as features, because the protein binding sites are more conserved than other surface residues [4]. Some kinds of conservation scores were proposed; a comprehensive evaluation of these scores was reported in [5]. One of the most widely used features is the Accessible Surface Area (ASA) [4], because the binding sites show higher ASA values than those of the other surface residues [6].

Some machine learning methods treated protein binding site prediction as a binary classification task, and some well-known machine learning techniques have been applied to this field, such as support vector machine [7, 8], neural network [1], Bayesian network [9], and hidden Markov model [10]. A comparison of these methods has been performed by Zhou and Qin [11].

In our previous study [12], we introduced a novel profile-level propensity for protein binding site prediction. Experimental results showed that this propensity can significantly improve the performance of the SVM based methods. Recently, we applied hidden Markov support vector machine (HM-SVM) to this field [13], which takes protein binding site prediction as a sequence-labeling task. The advantage of this method is that it is able to incorporate the sequence-order effects into the predictor. However, this method only uses two basic features (PSSM and ASA features) as input for protein binding site prediction. Therefore, it is interesting to explore whether the order profile propensity can improve the performance of HM-SVM based method or not. In this study, we proposed a computational method for protein binding site prediction by combining the hidden Markov support vector machine and the order profile propensity. When tested on six different data sets, the HM-SVM predictor using order profile propensity as an extra feature consistently outperformed the predictor only using two basic features (PSSM and ASA features); in particular, in terms of AUC, the performance is improved by more than 10 percent, indicating that combining the order profile propensity and the HM-SVM is a suitable approach to improve the accuracy of protein binding site prediction.

## 2. Methods

### 2.1. Dataset Description

**Figure 1:**
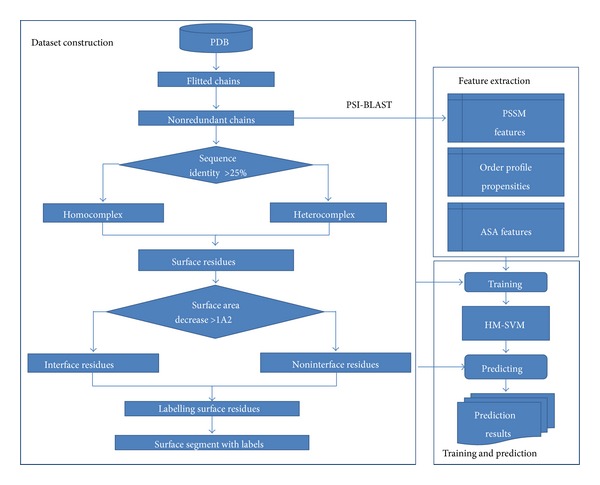
Overview of the proposed framework for protein binding site prediction.

The datasets used in this study have been described in the study [13]. 1124 protein chains were selected from the Protein Data Bank (PDB) [14]. The chains were divided into six types of datasets according to homology of interacting chains and the definition of the interface. The information of the six datasets is shown in Table 1, and the process of dataset preparation is shown in the left part of Figure 1. The six datasets can be downloaded from http://bioinformatics.hitsz.edu.cn/HMSVM-OP.

### 2.2. Feature Description

#### 2.2.1. Order Profile Propensity

The detailed information of how to calculate the order profile propensity was introduced in study [12]. Here we only briefly introduce this process. The order profile propensities were profile-based features, which extracted the evolutionary information from frequency profiles. The frequency profiles were calculated from the multiple sequence alignments outputted by running the PSI-BLAST software [13] searching against the nrdb90 database from EBI [15] with parameters of *j* = 10 and *e* = 0.001. The frequency profiles were converted into order profiles by combining the amino acids whose frequencies were higher than a given threadhold optimized on the benchmark dataset. Order profile can be viewed as a profile-based building block of proteins, which has been used for many tasks in the field of bioinformatics [12, 16].

The order profile propensity was based on the order profile occurrence differences between protein binding regions and other surface regions. The equations of how to calculate this feature were given by [12, Equations (3)–(5)].

#### 2.2.2. Position-Specific Score Matrix (PSSM)

PSSM was another profile-based feature, which was generated by using PSI-BLAST [13] with the parameters *j* and *e* set as 10 and 0.001, respectively.

#### 2.2.3. Accessible Surface Area (ASA)

We employed the DSSP program [17] to calculate the Accessible Surface Area (ASA) features, which were scaled by the nominal maximum area of each residue.

### 2.3. Hidden Markov Support Vector Machine

Hidden Markov support vector machine proposed by Altun et al. [15] was a sequential labelling model. In our previous study [13], it showed that when using the two basic features (PSSM and ASA features), the HM-SVM based method outperformed other machine learning methods, such as SVM, CRF, and ANN. In this study, we explored new features to improve the performance of HM-SVM based methods. For more information of HM-SVM, please refer to this paper [13].

The flowchart of the proposed computational method for protein binding site prediction was shown in Figure 1, in which the left part shows the process of dataset construction, and the right part shows the prediction process of the model based on HM-SVM.

In this paper, SVM^hmm^ toolkit (V3.10) was employed as the software of HM-SVM model with parameters *c* and *e* set as 0.1 and 1, respectively. This parameter combination was optimized with the training data. The input features of HM-SVM include order profile propensity, ASA, and PSSM. These features were extracted from the target residues and its 6 neighbouring residues in each direction.

### 2.4. Evaluation Methodology

The sensitivity (Sn), specificity (Sp), overall accuracy (Acc), F1 measure (F1), Matthews correlation coefficient MCC, and AUC can be, respectively, expressed as [18–22]

(1) Sn=TPTP+FN,Sp=TNTN+FP,Acc=TP+TNTP+FP+TN+FN,MCC =(TP∗TN)−(FN∗FP)(TP+FN)∗(TN+FP)∗(TP+FP)∗(TN+FN),AUC:  the  area  under  ROC  cure,

where TP represents the true positive, TN represents the true negative, FN represents the false negative, and FP represents the false positive.

## 3. Results 

In order to validate whether the order profile propensities can improve the performance of the HM-SVM based methods or not, two HM-SVM predictors with different features were constructed. The first HM-SVM employed the PSSMs and ASA as input features. This predictor was treated as a baseline predictor. For the second HM-SVM predictor, order profile propensity is added as an extra feature to evaluate whether this feature can improve the performance or not. The performance of the two HM-SVM predictors was evaluated by fivefold cross-validation.

The results of the two HM-SVM predictors on the six datasets are shown in Table 2. It can be seen that the first HM-SVM predictor using the two basic features achieved the lowest performance. The second HM-SVM predictor using the order profile propensity as an extra feature achieved the best performance on all the six data sets, especially its AUC score being about 10% higher than that of the first HM-SVM predictor, indicating that order profile propensity can significantly improve the performance of the HM-SVM based methods. In our previous study [13], we showed that the first HM-SVM predictor outperformed some state-of-the-art methods, such as ANN, CRF, and SVM. The second HM-SVM predictor significantly outperformed the first HM-SVM predictor, indicating that the proposed computational method for protein binding site prediction is a good method in this field.

Šikić et al. [23] proposed a method based on random forest, which was evaluated on a heterocomplex data set, and achieved good performance (Sp = 76.45%, Sn = 38.06%, F1 = 50.82%, and Acc = 80.05%). Our method (results of heterocomplex II dataset) outperformed this method by 14.98% in terms of F1, which further confirms the better performance of our method than some state-of-the-art methods.

## 4. Conclusion

In this study, we proposed a computational method for protein binding site prediction, which combines the order profile propensity and hidden Markov support vector machine. This method predicts the protein binding sites with a sequential labelling approach and uses a recently proposed feature to further improve the performance: order profile propensity, which contains the evolutionary information extracted from the sequence profiles. The main contribution of this study is that we validate the fact that order profile propensity can significantly improve the performance of the HM-SVM based method. The main advantage of the proposed method is that it treats the protein sequence as a whole and is able to use the label information of neighbour residues and the evolutionary information extracted from the frequency profiles. However, the order profile propensity was generated based on the frequency profiles, which require the computational expensive multiple sequences alignment process. It is the main disadvantage of the proposed method.

As noted by Li et al. [24], choosing proper features is a challenging task, especially for sequential labelling method, such as HM-SVM and conditional random field (CRF). In their experiments, the authors found that by simply adding some features into CRF cannot improve the performance of their method. Therefore, the obvious performance improvement when using order profile propensity as an extra feature will benefit our future studies, especially for the research on applying sequential method to this field. As pointed out in a comprehensive review and carried out in a series of recent publications [25–43], finding suitable features is the key step to improve the performance.

Furthermore, since user-friendly and publicly accessible web servers represent the future direction for developing practically more useful predictors [44, 45], we shall make efforts in our future work to provide a web server for the method presented in this paper.

## Figures and Tables

**Table 1:** Summary of six data sets.

Data set	Chains	Res.	Surface res.	Interface res.
Heterocomplex I^a^	504	109829	92797	26085
Homocomplex I	620	172917	141295	38170
Mix^b^ I	1124	282746	234092	64255
Heterocomplex II^c^	504	109829	92797	32386
Homocomplex II	620	172917	141295	45633
Mix II	1124	282746	234092	78019

^a^Type I data set with minor interface as negative samples.

^b^The mixed data set of heterocomplexes and homocomplexes.

^c^Type II data set with minor interface as positive samples.

**Table 2:** Performance of HM-SVM based method with and without order profile propensities.

Dataset	Method	Sp %	Sn %	F1 %	Acc %	MCC	AUC %
Heterocomplex I	HM-SVM 1^a^	44.9	56.0	49.8	68.3	0.274	69.5
	HM-SVM 2^b^	**52.4**	**73.5**	**61.2**	**73.8**	**0.436**	**81.4**

Homocomplex I	HM-SVM 1	45.4	60.0	51.70	69.7	0.309	72.2
	HM-SVM 2	**54.5**	**74.6**	**62.9**	**76.3**	**0.474**	**83.6**

Mix I	HM-SVM 1	45.5	58.0	51.0	69.4	0.297	71.2
	HM-SVM 2	**53.5**	**74.0**	**62.1**	**75.0**	**0.455**	**82.5**

Heterocomplex II	HM-SVM 1	54.0	56.7	55.3	68.0	0.305	70.7
	HM-SVM 2	**60.8**	**71.7**	**65.8**	**74.0**	**0.454**	**81.2**

Homocomplex II	HM-SVM 1	53.3	60.1	56.5	70.1	0.340	73.4
	HM-SVM 2	**61.1**	**73.8**	**66.8**	**76.4**	**0.493**	**83.7**

Mix II	HM-SVM 1	53.6	58.6	56.0	69.3	0.326	72.4
	HM-SVM 2	**61.0**	**72.7**	**66.3**	**75.2**	**0.474**	**82.4**

^a^Results of HM-SVM 1 on the six data sets are obtained from [13]. HM-SVM 1 represents the HM-SVM predictor with the basic feature set using PSSM and ASA features; ^b^HM-SVM 2 represents the HM-SVM predictor with the feature set using PSSM, ASA, and order profile propensity features.
